# Geotechnical Engineering Properties of Cement Fly Ash Gravel Mixtures for Application as Column-Supported Highway and Railway Embankments

**DOI:** 10.3390/ma15113972

**Published:** 2022-06-02

**Authors:** Pornkasem Jongpradist, Krissakorn Krairan, Pitthaya Jamsawang, Xiaobin Chen

**Affiliations:** 1 Construction Innovations and Future Infrastructures Research Center, Department of Civil Engineering, Faculty of Engineering, King Mongkut’s University of Technology Thonburi, Bangkok 10140, Thailand; pornkasem.jon@kmutt.ac.th; 2 Soil Engineering Research Center, Department of Civil Engineering, King Mongkut’s University of Technology North Bangkok, Bangkok 10800, Thailand; krissakorn.kr@gmail.com; 3 Key Laboratory of Heavy-Haul Railway Engineering Structures, School of Civil Engineering, Central South University, Changsha 410017, China; chen_xiaobin@csu.edu.cn

**Keywords:** column, consolidation, highway and railway embankments, soil improvement, strength

## Abstract

Our study investigates the geotechnical engineering properties of cement fly ash gravel mixtures in the laboratory. Gravels with three different size ranges were blended with cement and fly ash. The mixture properties were investigated, including the porosity, density, permeability, unconfined compressive and splitting tensile strengths, cohesion, and friction angle after curing for 28, 50, and 90 days, respectively. The experimental results revealed that the gravel sizes and fly ash contents significantly influenced the strength characteristics. The permeability coefficients of the cement fly ash gravel mixtures were 0.9 to 1.7 cm/s, much higher than a soil-cement column. The unconfined compressive strengths and splitting tensile strengths were found to be from 3.75 to 18.5 MPa and from 0.5 to 2.5 MPa, respectively. The cohesion and friction angle values ranged from 2.2 to 5.3 MPa and 30 to 40 degrees. The mixture strength was 6 to 30 times higher than a soil-cement column. The 15% fly ash provided the best strength characteristics as it exhibited the most significant calcium silicate hydrate contents. Thus, using cement fly ash gravel column-supported embankments is more productive than using a soil-cement column and granular pile to increase the column-bearing capacity and overall stability and accelerate the consolidation process.

## 1. Introduction

**Figure 1:**
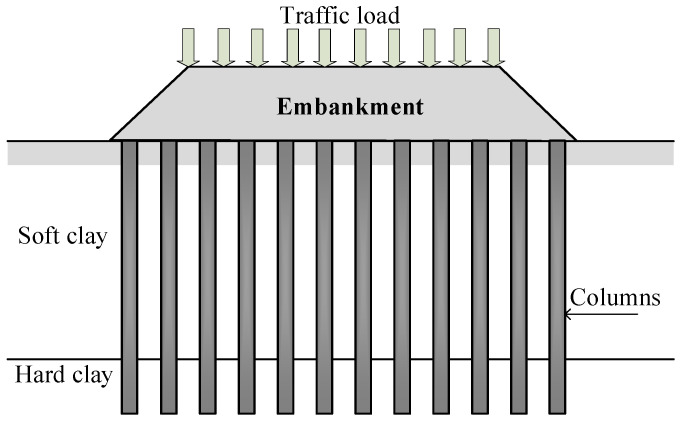
A typical cross-section of column-supported highway and railway embankments on a soft clay foundation.

**Figure 2:**
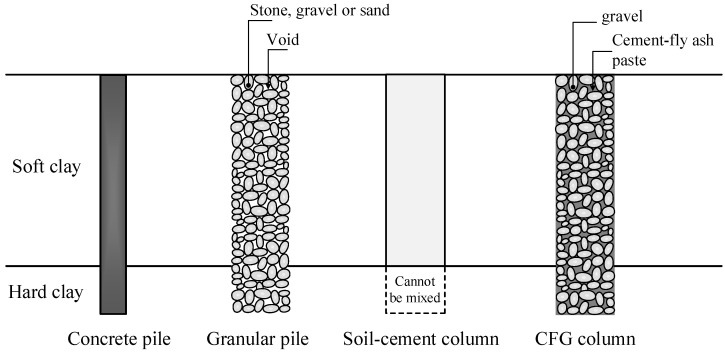
Various types of columns used for supporting the embankments on a soft clay foundation.

Since soft clay has a low shear strength, high compressibility, and low permeability, the construction of highway and railway embankments on the soft clay layer results in slope instability, low bearing capacity, and significant settlement problems. Such problems can create construction delays and additional rebuilding costs [1]. Moreover, consolidation settlement caused by dissipating excess pore water pressure takes a long period because of the low permeability of the soft clay. Therefore, a soil improvement technique, a column-supported embankment, has been commonly introduced to maintain slope stability, increase the bearing capacity, and reduce settlement [2], as shown in Figure 1. The columns used for supporting embankments built on soft clay foundations include concrete piles, granular columns (stone columns or sand compaction piles), and soil-cement columns (Figure 2). Concrete piles have high strength and can be driven through the stiff to hard clay layers to obtain high pile capacity and reduce embankment settlement. Granular piles are built by driving a steel casing to the hard clay layer from the ground surface. The clays contained in the case are removed and replaced with stones, gravels, or sands to enhance the bearing capacity of the soft clay [2]. The granular pile can accelerate the consolidation process induced in the soft clay since it acts as a vertical drain because of the high permeability of sand or stone used. The excess pore water pressure generated by the traffic and embankment loads can be rapidly dissipated through vastly interconnected pores between particles of sand and stone [2]. However, the primary disadvantage of granular piles is that the aggregates used to create the columns are cohesionless materials without internal bonds. The granular pile’s typical cohesion and friction angle values ranged from 1 to 10 kPa and 30 to 40°, respectively [2,3,4]. The perpendicular stress from the embankments is required to produce shear strength. Therefore, the granular piles are ineffective under low vertical stress situations, including those located at the embankment toe and granular piles used for lateral support work in soft clay.

The soil-cement column technique is a possible method to avoid such problems. This technique has the advantage of rapid construction since it involves the in situ mixing of soft clay and cement power or cement slurry [5,6,7]. The cement-treated soft clay has high internal bonds between clay particles induced by cement hydration products during chemical reactions. The soil-cement columns have a high unconfined compressive strength from 0.6 to 2 MPa without requiring high perpendicular stress like the granular piles [8,9]. For a high embankment, the embedment of the soil-cement columns in hard clay (Figure 2) is needed to derive the fixity situation, increasing the column-bearing capacity and overall stability [1]. Since the soil-cement columns have low tensile strength, tensile or flexural failure can occur for columns located at the embankment toe [10,11]. Although the concrete pile has a much higher strength than the granular pile and soil-cement column, the concrete pile is also more expensive due to the material cost. Thus, the concrete pile is unsuitable for supporting the embankment in terms of cost-effectiveness. 

Pervious concrete is a particular type of concrete with a high porosity utilized for concrete flatwork applications allowing water to pass directly through, such as parking and light traffic areas, residential streets, and greenhouses [12]. Pervious concrete consists of cement, large coarse aggregate, and water with little to no fine aggregates, and the mixture has a water-to-cement ratio of 0.28 to 0.40 [12,13]. The admixture and aggregate characteristics significantly affect porous concrete’s strength and porosity [14,15,16,17,18,19,20,21,22]. The pervious concrete consists of cement, large coarse aggregate, and water with little to no fine aggregates and has a common unconfined compressive strength of 2.8 to 28 MPa [12] up to 50 MPa [14]. However, the high-performance pervious concrete exhibits a compressive strength of 15 to 65 MPa [15,17]. The Portland cement pervious concrete’s tensile strength falls between 0.2 and 2.4 MPa and 1 and 10.4 MPa, respectively, corresponding to the tensile strength to compressive strength ratio of 0.14–0.17. [16,22]. The elastic modulus of high-performance pervious concrete is 26 to 41 GPa and increases with increasing compressive strength [17]. The pervious concrete has a porosity value of 18 to 35% [12], contributing to flowability [14,15,16,17,18,19,20,21,22]. The permeability coefficient of pervious concrete is between 0.01 and 14 cm/s [14,15,16,17,18,19,20,21,22], which is high enough to be considered a porous material. Standard concrete’s cohesion and friction angle values obtained from triaxial compression tests were 5–19 MPa and 27–39°, respectively [23]. However, some of the rocks’ cohesion and friction angle values based on triaxial compression tests ranged from 4.5 to 36 MPa and 28 to 45°, respectively [24]. Although the strengths of pervious concrete are higher than the granular pile and soil-cement column, this type is an improper column to support highway and railway embankments. Its strength surpasses the required values, leading to high construction costs.

Fly ash is a byproduct of coal power plants, and its production rate is higher than the rates of recycling and reuse [25,26]. Even though fly ash generates a slower hydration reaction than cement in concrete, it provides notable environmental advantages, such as relieving air and reducing water pollution [27]. Controlled low strength material, also known as flowable fill, is a weak, runny concrete mix. It is considered impervious concrete used in construction for non-structural purposes such as backfill or road bases. Controlled low strength material consists of fly ash, cement, sand, water, and 8–25% entrained air and has a strength of less than 8.3 MPa [28]. The replacement of cement with 35% fly ash provided a desirable compressive strength of the recycled aggregate concrete. Reduced strength is observed for fly ash replacement levels > 35% [29]. The recycled aggregate concrete containing fly ash exhibits higher strength than sole cement concrete [29] because the fineness of the ground fly ash particle can fill the voids between the cement and aggregates. Moreover, fly ash expedites the pozzolanic reaction, producing additional calcium silicate hydrates. Thus, interfacial bonding between the aggregates and pastes was improved, resulting in the increased strength of the cement–fly ash concrete [29,30,31]. 

The current study introduces cement fly ash gravel (CFG) mixtures created by mixing cement, fly ash, and gravel as a column to support embankments built on soft clay instead of the concrete pile, granular pile, or soil-cement column. The cement was partially replaced with fly ash to save the cement cost and relieve environmental problems related to using cement alone [26]. Thus, the raw materials used for the CFG mixture utilizes are different from pervious concrete and controlled low strength material. Moreover, using the CFG mixture in civil engineering works differs from pervious concrete and controlled low strength material, as earlier mentioned. The CFG column exhibits high bonding and can be embedded in the stiff to hard clay layer (Figure 2), employing a similar construction method to a granular pile. The strength of the CFG column is sufficiently high to resist high embankment stress, and the material cost of the CFG column is safer than porous concrete and regular concrete. 

**Figure 3:**
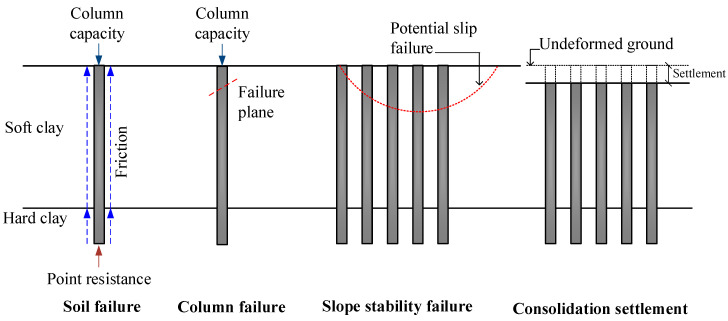
Various failure modes and settlement of a CFG column-supported embankment built on soft clay.

The bearing capacity of a single CFG column subjected to embankment loads, as shown in Figure 3, is governed either by the shear strength of the soil (soil failure) or by the strength of the CFG column (column failure). The soil failure depends on both the skin friction resistance of the column and the point resistance, while the column failure depends on the unconfined compressive strength of the CFG mixture. The slope stability failure of the CFG column-improved soft clay depends on the shear strengths of CFG columns and unimproved soft clay. The shear strength parameters required for slope stability calculation include internal friction angle and cohesion. Moreover, the embankment and traffic loads can induce consolidation settlement of the CFG column-improved soft clay. However, limited previous experimental studies on the geotechnical properties of CFG mixtures using various gravel sizes and fly ash replacement levels have been performed. In our study, the studied properties of the CFG mixtures were porosity, density, permeability coefficient, unconfined compressive and tensile strengths, elastic modulus, cohesion, and internal friction angle. All samples were cured at 28, 50, and 90 days before testing. 

## 2. Materials and Methods

### 2.1. Materials 

**Figure 4:**
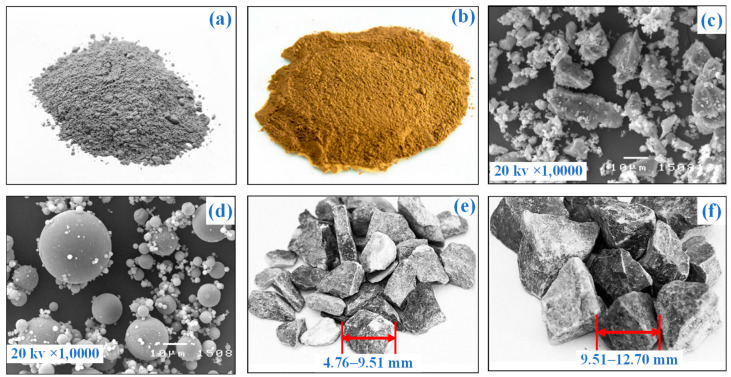
(**a**) Cement, (**b**) fly ash, (**c**) SEM photos of cement and (**d**) fly ash particles; (**e**) small gravel, and (**f**) large gravel used in this study.

The type I ordinary Portland cement type and fly ash, as shown in Figure 4a,b was used as cementitious and pozzolanic materials for the current study. Fly ash was obtained from an electric power plant, which is located in the Lampang province of Thailand. Photos obtained from scanning electron microscopy (SEM) techniques in Figure 4c,d reveal that cement particles exhibited rough surfaces and nonuniform angular shapes, whereas the fly ash particles showed spherical shapes with uneven surfaces. The fly ash has a specific gravity of 2.53 and fineness of 3200 to 3600 cm^2^/g. Table 1 shows the chemical composition of cement and fly ash based on X-ray fluorescence analysis and illustrates that CaO and SiO_2_ were the primary compounds of cement and fly ash, respectively. Fly ash contains combinations of SiO_2_, Al_2_O_3_, and Fe_2_O_3_ between 50 and 70%; thus, the fly ash used in this study is considered class C following ASTM standard C 618 [32]. The class-C fly ash has both pozzolanic and cementitious properties because its high CaO amount, 17.85%, is more effective than class F fly ash. In addition to being a good pozzolan, class-C fly ash is more available and cheaper than other binders in Thailand, such as blast furnace slag, red mud, and metakaolin. Thus, class-C fly ash was chosen as a supplementary cementitious material for this study.

Limestone gravels were derived from Saraburi province, Thailand. This province is a primary source for supplying the natural gravel for construction materials. Gravels of three different sizes were employed as coarse aggregates to produce CFG mixtures, including small (SG), mixed gravel (MG), and large gravel (LG). The SG and MG have size ranges of 4.75–9.5 mm and 9.5–19.5 mm, as shown in Figure 4e,f, respectively. The MG comprises a combination of 50% small gravel and 50% large gravel. The gravel particles are rough and sharp, showing good strength characteristics. The gravel properties are listed in Table 2.

### 2.2. Specimen Preparations 

**Figure 5:**
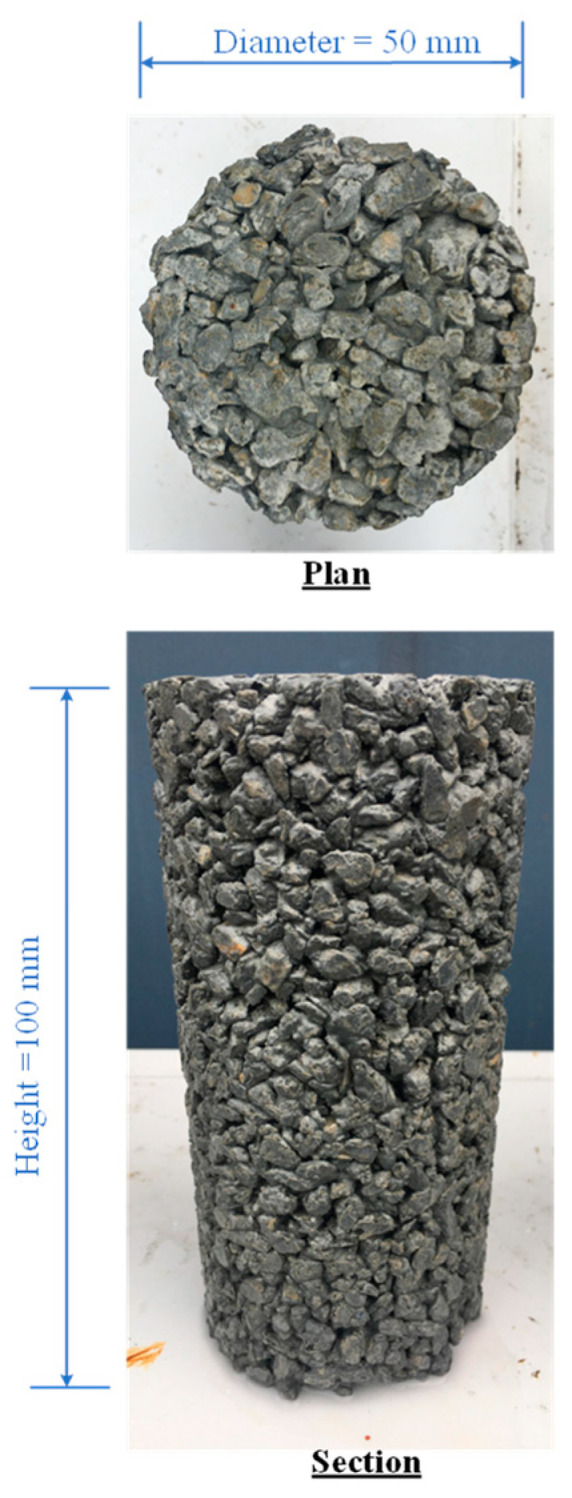
CFG specimen.

Since the cylindrical sample used in the triaxial compression test had a diameter of 50 mm and a height of 100 mm, the same sample size was used to determine density, porosity, and permeability coefficient values and compressive and splitting tensile strength tests to avoid the sample size effect. The cement contents were partially replaced with fly ash at levels of 5 to 25% by cement weight. The water–cement ratio was fixed to 0.32, and the curing periods were 28, 50, and 90 days. The mixture was prepared sufficiently for one sample only to control the mixing quality. The CFG mixture proportions are listed in Table 3. The gravel, cement, and fly in a concrete mixer first mixed ash for 2 min; then the water was added, and the mixture was blended for 3 min. The uniformity and homogeneity of the specimen were satisfied by visual observation. Each sample was contained in a cylindrical steel mold, which could be split into two parts to avoid the specimen disturbance and was greased with lubrication oil. After completing the molding process for 24 h, the specimen was immediately removed. The mass and size of the sample were recorded before curing in the water bath. Figure 5 shows the CFG specimen before testing.

### 2.3. Methods

This section describes the testing details and procedures of tests. When the assigned curing time of the specimen for each test was achieved, the test was then instantly performed. The acceptance criterion was designated that the individual test values of three samples, molded with the same characteristics, must deviate from the mean test value by less than 10% to avoid the error caused by the discreteness of the sample. For all tests in this study, an average value of the test results based on three specimens was reported.

#### 2.3.1. Density and Porosity

The density (*ρ*) and porosity (*n*) of the CFG samples were determined following the ASTM C1754 standard test method for the density and void content of hardened porous concrete [33]. The specimen was oven-dried at a temperature of 38 °C for 24 h, and the dry mass was subsequently measured after removing it from the oven. The ρ and n were calculated as follows:

(1) $$\rho =\frac{M_{d}}{V_{\mathrm{avg}}}$$

where:

*M*_d_ = dry mass of the specimen (kg)
*V*_avg_ = average volume of the sample (m^3^)

(2) $$n=\left[1-\left(\frac{M_{d}-M_{\mathrm{sub}}}{\rho_{w}V_{\mathrm{avg}}}\right)\right]\times 100\%$$

where:

*M*_sub_ = submerged mass of the specimen (kg)
*ρ*_w_ = density of water at temperature of the water bath (kg/m^3^)

**Figure 6:**
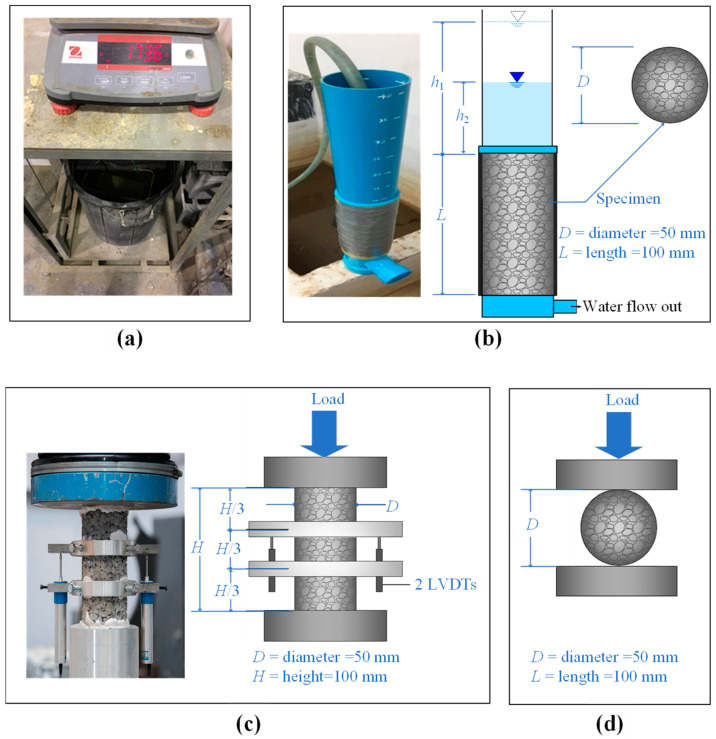
Test apparatus for determining (**a**) submerged density, (**b**) permeability coefficient, (**c**) unconfined compressive strength, and (**d**) splitting tensile strength.

The test apparatus for measuring the submerged mass of the specimen is presented in Figure 6a.

#### 2.3.2. Permeability Coefficient

The permeability coefficient of the CFG sample was determined following the ASTM D2434 standard test method for the permeability of granular soils [34] by adapting the falling head test method. The test apparatus is illustrated in Figure 6b. The CFG sample was placed in a cylindrical plastic tube. The tube was tight to prevent water leakage along the sides of the sample. The small gap between the specimen and tube at the bottom was sealed to avoid water infiltration through the edge of the tube. The permeability coefficient rate of porous concrete was subsequently calculated by Equation (3).

(3) $$k=\frac{aL}{At}\times \ln\frac{h_{1}}{h_{2}}$$

where:

*k* = permeability coefficient of the CFG sample (cm/s)
*a* = area of the cylindrical tube (cm^2^)
*A* = area of the specimen (cm^2^)
*L* = length of the sample (cm)
*t* = time for water to pass from level *h*_1_ to *h*_2_ (s) through the tube

#### 2.3.3. Unconfined Compression and Splitting Tension Tests

Before testing, the stone cap was used to cover both ends of the specimen to ensure that the end part was flat. The unconfined compressive strength (*q_u_*) and splitting tensile strength (*q_t_*) were determined according to the ASTM C39 [35] and ASTM C496/C496M-17 [36], respectively. Both tests were performed on the specimens using the automatic loading machine with a capacity of 100 kN (Figure 6c,d). Vertical stresses with a stress rate of 0.25 MPa/s were applied to the specimen until failure. Two 50-mm capacity linear variable differential transducers (LVDTs) were used to determine the average vertical displacement of the sample in unconfined compression tests. This setup helps to calculate the *q_u_* and elasticity modulus (*E*_50_), which is defined as the secant modulus at 50% of the *q_u_*. The *q_u_* was taken to be the maximum compressive stress. The *E*_50_ of each sample could be estimated from the slope of the stress-strain curve obtained from the unconfined compression test.

#### 2.3.4. Triaxial Compression Test

**Figure 7:**
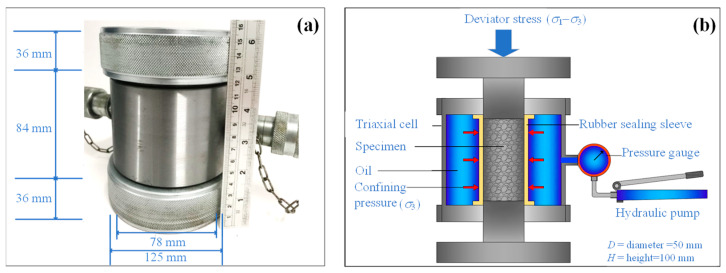
Triaxial test apparatus, including (**a**) Hoek’s cell and (**b**) test setup.

**Figure 8:**
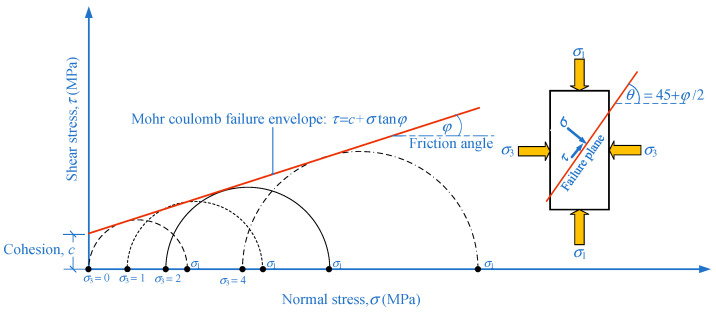
Mohr-Coulomb failure envelope for CFG the samples under triaxial compression tests.

The triaxial compression test was performed according to the ASTM D2664-95a test [37] for determining shear strength parameters, including cohesion (*c*) and internal friction angle (*φ*) of the CFG specimen under triaxial stresses. The specimen was inserted into the rubber sealing sleeve. The covered sample was delivered into Hoek’s cell, as shown in Figure 7a,b. Our study used oil and hydraulic pumps to generate confine pressures. The confining pressure (*σ*_3_) applied to the sample ranges between 0, 1, 2, and 4 MPa. The universal testing machine with a capacity of 100 kN was used to apply the deviator stress (*σ*_1_ – *σ*_3_) to the specimen until failure with a stress rate of 0.15 MPa/s. The *c* and *φ* values of the CFG specimens were calculated based on the Mohr-Coulomb failure criterion. The major principal stress (*σ*_1_) can be taken as *σ*_1_ = *σ*_3_ + (*σ*_1_ – *σ*_3_), whereas the minor principal stress is equal to the confining pressure (*σ*_3_). Figure 8 shows an example of plotting the Mohr’s circles at failure obtained from triaxial tests for this study. The failure envelope can be obtained by drawing a line that touches all Mohr’s circles. The failure envelope is approximately a straight line intercepting the *y*-axis and can be expressed by the equation:

(4) τ=c+σtanφ

where:

*τ* = shear strength (MPa)
*σ* = normal stress on the failure plane (MPa)
*c* = cohesion of the CFG specimen (MPa)
*φ* = internal friction angle of the CFG specimen (degree)

## 3. Results and Discussion

### 3.1. Porosity

**Figure 9:**
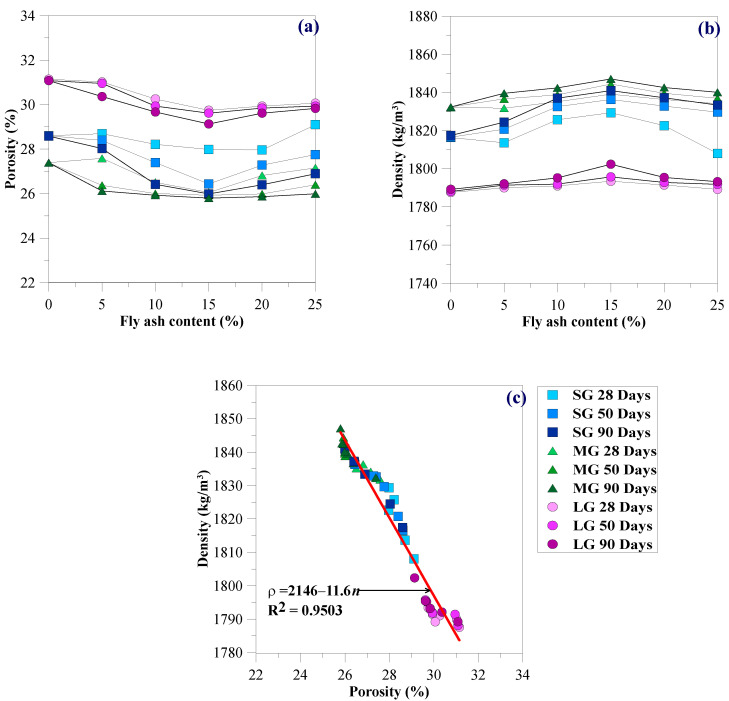
Relationships between (**a**) porosity and fly ash content, (**b**) density and fly ash content, and (**c**) density and porosity.

Figure 9a illustrates the effects of gravel size, fly ash content, and curing periods for the Porosity (*n*) of the CFG mixture. The *n* values for CFG mixtures vary between 25.9 and 31.2%, falling in the range of conventional porous concrete obtained from previous studies due to the same gravel size ranges [15,17,18,21,38]. The MG, containing various particle sizes, exhibited the best particle placement. Thus, the MG showed the smallest *n* values for the same fly ash contents and curing times. However, the LG with the largest internal pores between particles exhibited the greatest *n*. The SG, having smaller internal pores than LG and poorer particle size distribution than MG, showed *n* values falling between MG and LG. The *n* values decreased as the fly ash contents increased from 5 to 15%. However, since the fly ash content was more than 15%, the *n* values increased. Fly ash acts as a filling material like fine aggregates to fill voids between the gravel particles in the CFG mixtures, reducing the porosity. Fly ash is a pozzolan reacting with cement and water to form additional calcium silicate hydrates (CSH). The CSH expanded with curing time induced by pozzolanic reaction and could fill the CFG mixture’s pores. This phenomenon decreases the *n* values with increasing curing periods from 28 to 90 days [29]. Thus, 15% fly ash provided the best fulfillment of filling materials and pozzolanic reaction products in the CFG mixtures’ pores. 

### 3.2. Density

Figure 9b presents the effects of fly ash content, gravel size, and curing time on the density (*ρ*) of CFG mixtures. The *ρ* of the CFG mixtures ranged between 1780 and 1860 kg/m^3^, with an average value of 1820 kg/m^3^. The *ρ* was significantly influenced by fly ash content for any gravel size and curing time. The *ρ* increased proportionally with increasing fly ash content of 5 to 15%. The 15% fly ash provided the highest *ρ* because it exhibited the smallest *n*, as discussed in Section 3.1. The *ρ* of the CFG mixtures is smaller than standard concrete (2400 kg/m^3^) and falls in the range of porous concrete (1500 to 2000 kg/m^3^) [16,22]. The *ρ* of the CFG mixtures increased with increasing curing periods due to the pozzolanic reaction and was affected by gravel sizes due to the particle packing effect. As expected, the *ρ* of the CFG mixtures increased with decreasing *n*, as shown in Figure 9c. The *ρ*−*n* relationship obtained from the present research in Figure 9c illustrates the exponential decay function as follows: *ρ* (kg/m^3^) = 218e^−0.0064*n*(%)^.

### 3.3. Permeability Coefficient

**Figure 10:**
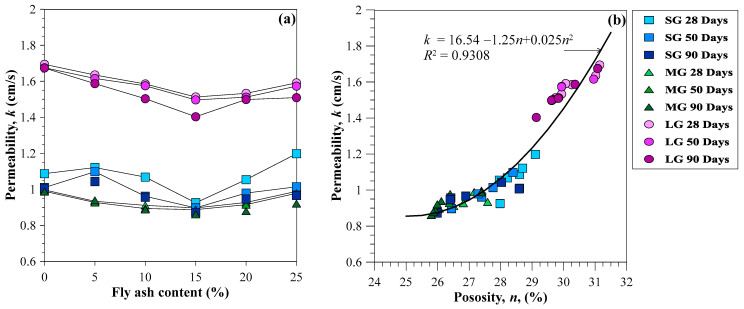
Relationships between (**a**) permeability coefficient and fly ash content and (**b**) permeability coefficient porosity.

Figure 10a shows that the permeability coefficient (*k*) and porosity (*n*) had similar characteristics. The *k* values decreased as the fly ash contents increased from 5 to 15%. However, since the fly ash content was more than 15%, the *k* values increased. The *k* values of the CFG mixtures varied between 0.9 and 1.7 cm/s. The LG gave the *k* values of 1.4 to 1.7 cm/s, which falls in clean gravel (>1.0 cm/s) [39]. However, the SG and MG provided *k* values of 0.9 and 1.2 cm/s, equivalent to clean sand, gravel-sand mixtures, and clean gravel. Therefore, CFG with LG can serve as an excellent drainage material, while CFG using SG and MG can be a good drainage material. Notably, the CFG with SG was more permeable than the CFG utilizing MG for use as a CFG column.

The CFG provided a lower k than the pervious concrete obtained by Bhutta et al. [21] by approximately 3–4 times due to using larger coarse aggregates than this study. However, the *k* values of CFG mixtures were higher than that of the high-performance pervious concrete using smaller aggregates with silica fume and silica powder studied by Zhong and Wille [17]. The *k* values for the pervious concrete utilizing gravel sizes and admixtures by Ibrahim et al. [16] were close to that for the CFG mixtures. Figure 10b illustrates the correlation between the *k* and *n* of CFG mixtures derived from this study. As expected, the *k* of the CFG mixtures increased with increasing n. The *ρ*−*n* correlation was modeled as a polynomial quadratic function as follows: *k* (cm/s) = 16.54 − 1.25*n* + 0.025*n*^2^. 

### 3.4. Unconfined Compressive Strength

**Figure 11:**
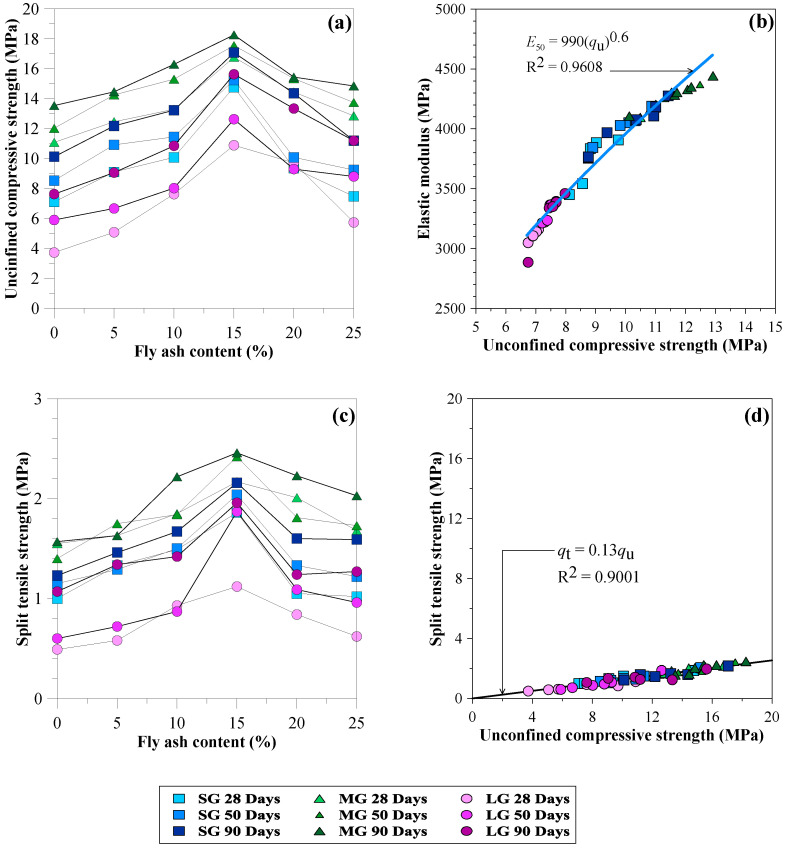
Relationships of (**a**) unconfined compressive strength versus fly ash content, (**b**) relationship of elastic modulus and unconfined compressive strength, (**c**) splitting tensile strength versus fly ash content, (**d**) relationship of splitting tensile strength and unconfined compressive strength.

Figure 11a presents the effects of fly ash content, curing period, and gravel size on the unconfined compressive strength (*q*_u_) of CFG mixtures. The *q*_u_ varied from 3.8 to 18.2 MPa. The average *q*_u_ value was 11 MPa, greater than the *q*_u_ of the soil-cement column (1 MPa) by 4–18 times. The fly ash content significantly impacted *q*_u_ for any gravel size and curing time. The *q*_u_ values increased proportionally as the fly ash content increased from 5 to 15%. The 15% fly ash content exhibited the highest *q*_u_ values, corresponding to the lowest *n* and greatest *ρ*, as described in Section 3.1 and Section 3.2. The *q*_u_ values were reduced in all curing periods as the fly ash content raised above 15%, according to other aggregates blended with cement and fly ash [29]. For example, the *q*_u_ values at 28 days for MG-F0 and MG-F15 were 11.0 and 16.5 MPa, respectively. The *q*_u_ value at 90 days for MG-F15 was 18.2 MPa. Thus, replacing cement with 15% fly ash increased the *q*_u_ by 65%.

Fly ash acts as a filling material to fill voids between the cement and gravel particles in the CFG mixtures, reducing the porosity. The class-C fly ash with high reactivity also acts as a pozzolanic material because SiO_2_ and Al_2_O_3_ react with calcium hydroxide (CaOH_2_) generated by the cement hydration process to form additional calcium silicate hydrates (CSH) [29,30,31]. These characteristics decrease the porosity and increase the strength of the CFG mixtures with increasing curing periods from 28 to 90 days [29,30,31]. Even though the substitution rate of fly ash increased, the porosity decreased. Therefore, the *q*_u_ values for CFG mixtures without fly ash, including SG-F0, MG-F0, and LG-F0, were lower than the *q*_u_ values for CFG mixtures with fly ash at all curing periods.

Regarding 15% fly ash for 28 days, the MG-F15 exhibited the highest *q*_u_ value at 16.5 MPa. However, the SG-F15 and LG-F15 revealed reducing *q*_u_ values of 7.0 and 3.8 MPa, lower than MG-F15 by 58 and 77%, respectively. Therefore, the gravel sizes significantly affected the *q*_u_ values of the CFG mixtures. It can be concluded that 15% fly ash provided the best fulfillment of filling materials and pozzolanic reaction products in the CFG mixtures’ pores, resulting in the greatest strength.

### 3.5. Elasticity Modulus

Figure 11b presents the relationship between the *E*_50_ and *q_u_* of CFG mixtures. The variations in *E*_50_ were 2900 to 4400 MPa, corresponding to *q_u_* values of 6.7 to 12.9 MPa. The *E*_50_ value of the CFG mixtures is approximately nine times lower than that of high-performance pervious concrete [17], ranging from 26 to 41 GPa because of the larger voids in the CFG mixture. 

### 3.6. Splitting Tensile Strength 

Figure 11c shows the splitting tensile strength (*q*_t_) characteristics of the CFG mixtures. The CFG samples showed *q*_t_ = 10–14%*q*_u_. The MG-F15 exhibited the highest *q*_t_ values of 2.2 MPa and 2.5 MPa at 28 and 90 days, respectively. Thus, the *q*_t_ increased by approximately 14% due to the curing effect. The *q*_t_ values of CFG mixtures reported in this study are close to the *q*_t_ values of the Portland cement pervious concrete using similar gravel sizes between 4.5 and 12.5 mm and water–cement ratios of 0.30 to 0.40 revealed by Ibrahim et al. [16] and Joshaghani et al. [22]. The relationship between *q*_t_ and *q*_u_ in Figure 11d shows that *q*_t_ values are dependent on *q*_u_ values. As the *q*_u_ increases, the *q*_t_ also linearly increases. The relationship is expressed as a linear function: *q*_t_ = 0.12*q*_u_. 

### 3.7. Cohesion and Internal Friction Angle

**Figure 12:**
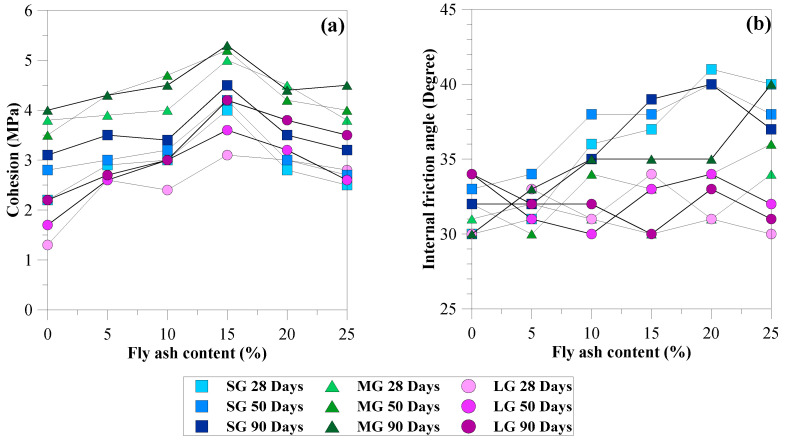
Relationships of (**a**) cohesion and fly ash content, (**b**) internal friction angle and fly ash content.

Figure 12a,b show the influence of fly ash content, curing period, and gravel size on the *c* and *φ* values of the CFG mixtures. The characteristics of *c* and *q*_u_ are similar because these shear strength parameters are related to the internal bonds of cement–fly ash paste in CFG mixtures. Unlike the characteristics of *φ*, this shear strength parameter depends on the overall friction of the materials used in CFG mixtures, including cement–fly ash paste, fly ash, and gravel, and examines the different failure planes. Therefore, the *φ* values are different in each mixture proportion and independent of the curing periods. However, some factors affecting the failure planes of the CFG samples include the homogeneity and uniformity of mixtures induced by mixing various materials. These factors provide the scatter results, as illustrated in Figure 12b, and thus no clear trend of internal friction characteristics for the CFG mixtures was observed. 

The *c* values varied from 1.4 to 5.4 MPa, with an average of 3.4 MPa, which is greater than the cohesion of the soil-cement column (0.5 MPa) [8,9] by 2.8–10 times. Öztekin et al. [23] determined *c* and *φ* values based on triaxial compression tests for normal concrete samples using aggregate sizes between 4–16 mm, cement contents of 350–500 kg/m^3^, and water–cement ratios of 0.30–0.60, providing the compressive strength values of 22–53 MPa. The *c* and *φ* values fell between 5.2 and 12.8 MPa and 27 to 34°, respectively. Yu et al. [40] performed a triaxial compression test on the porous cement concrete specimens utilizing the aggregate sizes of 4.75–9.5, cement contents of 1560–1700 km/m^3^, aggregate contents of 100–125 km/m^3,^ and water amount of 340–415 km/m^3^. The results showed that the porous cement concrete samples had a porosity of 20% and exhibited compressive strength values of 21–32 MPa. The *c* and *φ* values varied from 5.2 to 12.8 MPa and 38 to 42°, respectively.

Öztekin et al. [23] and Yu et al. [40] concluded that the *c* values depended on the compressive strength, whereas the *φ* values depended on aggregates’ gradation. Therefore, the *c* values of the CFG mixtures were smaller than normal concrete (5.2–12.8 MPa) [23] and porous cement concrete (5.2–12.8 MPa) [40] due to significant differences in compressive strength. Replacing cement with 15% fly ash exhibited maximum *c* values for all gravel sizes and curing times. The MG samples showed the greatest *c* values compared with SG and LG samples. The *φ* values of the CFG mixtures varied between 30 and 42°, falling in the range of the internal friction angle of gravel used as the stone column (30–40°) [2] normal concrete (27–34°) [23], and porous cement concrete (38–42°) [40]. Thus, the CFG column-bearing capacity and CFG column improved soft clay could be more than the stone column due to the high cohesion of the CFG mixtures. By contrast, those could be greater than the soil-cement column (zero friction angle) because of the higher cohesion and friction angle of the CFG mixtures. 

### 3.8. Failure Modes of the CFG Sample

**Figure 13:**
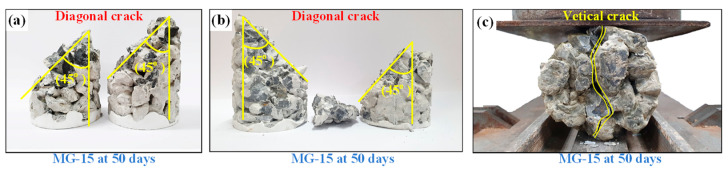
Typical failure modes of CFG specimens in the (**a**) unconfined compression, (**b**) triaxial compression, and (**c**) splitting tensile tests.

Figure 13a–c show the typical failure modes of the MG-15 samples subjected to unconfined compression and triaxial compression and splitting tension tests, respectively. The CFG samples’ diagonal shear fracture mode was detected under unconfined and triaxial compression tests, as shown in Figure 9a,b, respectively. The primary fractures were revealed in the cement–fly ash paste and the interfacial gravel and cement–fly ash paste. No fractures in the gravel body were observed since its strength is higher than the cement–fly ash paste. The failure planes containing the most considerable void, the weakest plane of the mixtures, were detected [41]. Thus, the strength of the CFG paste and void spread were two primary parameters influencing the failure mode of CFG samples. The failure mode affects the strength of various concrete types [19]. The general failure mode of a regular concrete sample is well-formed cones [42], causing higher strength than the CFG sample. The failure mode of CFG samples subjected to a splitting tension test was a single vertical tensile crack in CFG samples passing through interfacial gravel and cement–fly ash [43], as shown in Figure 13c. A single crack was observed in the middle of the cylindrical CFG sample, the weakest plane containing large voids [44,45]. 

## 4. Conclusions

Our research presents cement fly ash gravel (CFG) mixtures for use as column-supported highway and railway embankments built on a soft clay foundation. The geotechnical properties of CFG mixtures were experimentally investigated. Based on the results of our study, the following conclusions can be drawn:

1. Porosity is the primary factor governing the geotechnical properties of the CFG mixtures. The gravel size and cement significantly influenced the porosity–fly ash paste properties, depending on the curing period and fly ash content. The gravel containing a wide size range had the best particle packing, resulting in minor porosity and high strength.
2. The CFG mixtures had much higher permeability than the soil-cement columns
3. The unconfined compressive strength and cohesion of the CFG mixture are 3–13 times greater than that of the soil-cement column. By contrast, the internal friction angle of the CFG mixture is similar to the granular pile or stone column.
4. The cohesion and unconfined compressive strength characteristics are similar because these shear strength parameters are related to the internal bonds of cement–fly ash paste in the CFG mixtures. By contrast, the internal friction angle characteristics depend on the overall friction of the materials used in the CFG mixtures.
5. The CFG column capacity and CFG column-improved soft clay can be more than the stone column due to the high cohesion and friction angle of the CFG mixtures, which is higher than the soil-cement column with its zero-friction angle.
6. The cement replacement with 15% fly ash indicated the greatest strength and minor Porosity since 15% fly ash contributed the best void filling and proper portions of silicon dioxide and calcium hydroxide to produce a considerable amount of hydration and pozzolanic reaction products to fill the voids.
7. Using a cement fly ash gravel column for supporting embankments constructed on soft clay was more effective than using a soil-cement column and granular pile to enhance column-bearing capacity and the overall stability, reduce settlement and accelerate the consolidation process of the improved soft clay due to higher strength, stiffness, and permeability of fly ash gravel columns.

## Figures and Tables

**Table 1:** Chemical compositions of type 1 ordinary Portland cement and class-C fly ash used.

Compound	Cement	Fly ash
	(%)	(%)
CaO	62.81	17.85
SiO_2_	21.20	37.34
Al_2_O_3_	4.95	18.63
Fe_2_O_3_	2.82	13.17
Other	8.22	13.01

**Table 2:** Summary of gravel aggregate properties.

Test Description	SL	MG	LG
	4.76–9.51 mm	4.76–12.70 mm	9.51–12.70 mm
Absorption (%)	1.41	1.47	1.62
Specific gravity	2.61	2.61	2.61
Bulk specific gravity	2.26	2.29	2.23
Bulk density (kg/m^3^)	1535	1562	1505
Los Angeles abrasion (%)	17.5	16.2	15.4

**Table 3:** Mixture proportions for one cylinder of the CFG column.

Designation	SG	LG	Cement		Fly ash		Water
	(g)	(g)	(%)	(g)	(%)	(g)	(g)
SG-F0	400	-	100	88.0	0	-	28.16
SG-F5	400	-	95	83.6	5	4.4	
SG-F10	400	-	90	79.2	10	8.8	
SG-F15	400	-	85	74.8	15	13.2	
SG-F20	400	-	80	70.4	20	17.6	
SG-F25	400	-	75	66.0	25	22.0	
LG-F0	-	400	100	88.0	0	-	
LG-F5	-	400	95	83.6	5	4.4	
LG-F10	-	400	90	79.2	10	8.8	
LG-F15	-	400	85	74.8	15	13.2	
LG-F20	-	400	80	70.4	20	17.6	
LG-F25	-	400	75	66.0	25	22.0	
MG-F0	200	200	100	88.0	0	-	
MG-F5	200	200	95	83.6	5	4.4	
MG-F10	200	200	90	79.2	10	8.8	
MG-F15	200	200	85	74.8	15	13.2	
MG-F20	200	200	80	70.4	20	17.6	
MG-F25	200	200	75	66.0	25	22.0	

## Data Availability

Not applicable.
